# cMET inhibitor crizotinib impairs angiogenesis and reduces tumor burden in the C3(1)-Tag model of basal-like breast cancer

**DOI:** 10.1186/s40064-016-1920-3

**Published:** 2016-03-19

**Authors:** Alyssa J. Cozzo, Sneha Sundaram, Ottavia Zattra, Yuanyuan Qin, Alex J. Freemerman, Luma Essaid, David B. Darr, Stephanie A. Montgomery, Kirk K. McNaughton, J. Ashley Ezzell, Joseph A. Galanko, Melissa A. Troester, Liza Makowski

**Affiliations:** Department of Nutrition, University of North Carolina at Chapel Hill, Chapel Hill, NC 27599 USA; Department of Cell Biology and Physiology, University of North Carolina at Chapel Hill, Chapel Hill, NC 27599 USA; Lineberger Comprehensive Cancer Center, University of North Carolina at Chapel Hill, Chapel Hill, NC 27599 USA; Nutrition Obesity Research Center, University of North Carolina at Chapel Hill, Chapel Hill, NC 27599 USA; Department of Epidemiology, University of North Carolina at Chapel Hill, Chapel Hill, NC 27599 USA; Department of Pathology and Laboratory Medicine, University of North Carolina at Chapel Hill, Chapel Hill, NC 27599 USA

**Keywords:** Hepatocyte growth factor, Triple negative breast cancer, Obesity, Microenvironment, Microvascular density

## Abstract

Epidemiologic studies have associated obesity with increased risk of the aggressive basal-like breast cancer (BBC) subtype. Hepatocyte growth factor (HGF) signaling through its receptor, cMET, is elevated in obesity and is a pro-tumorigenic pathway strongly associated with BBC. We previously reported that high fat diet (HFD) elevated HGF, cMET, and phospho-cMET in normal mammary gland, with accelerated tumor development, compared to low fat diet (LFD)-fed lean controls in a murine model of BBC. We also showed that weight loss resulted in a significant reversal of HFD-induced effects on latency and elevation of HGF/cMET signaling in normal mammary and cMET in normal mammary and tumors. Here, we sought to inhibit BBC tumor progression in LFD- and HFD-fed C3(1)-Tag BBC mice using a small molecule cMET inhibitor, and began crizotinib treatment (50 mg/kg body weight by oral gavage) upon identification of the first palpable tumor. We next investigated if administering crizotinib in a window prior to tumor development would inhibit or delay BBC tumorigenesis. *Treatment:* Crizotinib significantly reduced mean tumor burden by 27.96 and 37.29 %, and mean tumor vascularity by 35.04 and 33.52 %, in our LFD- and HFD-fed C3(1)-Tag BBC mice, respectively. *Prevention:* Crizotinib significantly accelerated primary tumor progression in both diet groups but had no effect on total tumor progression or total tumor burden. In sum, cMET inhibition by crizotinib limited tumor development and microvascular density in basal-like tumor-bearing mice but did not appear to be an effective preventive agent for BBC.

**Electronic supplementary material:**

The online version of this article (doi:10.1186/s40064-016-1920-3) contains supplementary material, which is available to authorized users.

## Background

Basal-like breast cancer (BBC) accounts for 15–20 % of total breast cancers, with a higher prevalence in young and minority women such as African Americans and Hispanics (Carey et al. 2006; Boyle 2012). BBC is typically estrogen receptor (ER), progesterone receptor (PR) and human epidermal growth factor receptor-2 (HER2) negative (so called “triple-negative”) and is highly aggressive, exhibiting an early pattern of metastasis and poor overall prognosis. Thus, BBC presents a formidable challenge, as it lacks the molecular targets for current targeted drug treatments. High body mass index (BMI) is associated with poorer prognosis in breast cancer patients, including increased risk of lymph node metastasis, vascular invasion, disease recurrence, and mortality (Dawood et al. 2008; Calle et al. 2003; Biglia et al. 2013). Epidemiologic studies indicate that obesity is strongly associated with the BBC subtype in both pre- and post-menopausal women (Biglia et al. 2013; Boyle 2012). In obesity, rapid expansion in mammary adipose tissue leads to alterations in the stroma that mediate normal and tumor microenvironment dysfunction yet are poorly understood in breast cancer risk and progression (Iyengar et al. 2015; Ford et al. 2013; Johnson et al. 2012; Sundaram et al. 2013b). Given that obesity has increasing prevalence and is one of few modifiable risk factors for breast cancer, it is important to better elucidate the mechanisms for this obesity-associated cancer.

We have shown that BBC is characterized by unique epithelial-stromal interactions, which likely play a role in BBC etiology (Camp et al. 2011; Casbas-Hernandez et al. 2013; Brauer et al. 2013; Stewart et al. 2012). An elevated level of hepatocyte growth factor (HGF), a pleiotropic growth factor that signals through the receptor tyrosine kinase cMET, is characteristic of BBC. Elevation of HGF is also seen in obese patients (Bell et al. 2006). HGF/cMET signaling initiates an invasive growth program that promotes cell migration, invasion, proliferation, and angiogenesis (Mizuno and Nakamura 2013). Endothelial cell upregulation of cMET has been attributed to inherent or acquired resistance to antiangiogenic therapies targeting vascular endothelial growth factor (VEGF) in patients (Shojaei et al. 2010; Ding et al. 2003). Marking the first work in preclinical models paralleling human epidemiologic BBC findings, we used C3(1)-TAg mice, a unique genetically engineered mouse model (GEMM) of spontaneous BBC, to demonstrate that high fat diet (HFD)-induced obesity accelerated onset of tumor development and increased tumor aggressiveness as compared to low fat diet (LFD)-fed lean controls (Sundaram et al. 2013a). HFD also increased mammary gland HGF concentration and enhanced expression and activation of cMET. Using primary murine fibroblasts isolated from mammary glands or tumors, we further reported that obesity increased HGF production by mammary gland normal- and cancer-associated fibroblasts (NAF and CAF) (Sundaram et al. 2013a). Through signaling inhibition via an HGF blocking antibody, we showed that obese CAF-induced epithelial cell migration occurred in an HGF-dependent mechanism. Furthermore, using the intervention strategy of weight loss *prior to tumor latency*, we reported that weight loss blunted effects of HFD-induced obesity on multiple tumor parameters compared to mice maintained on HFD. Importantly, HFD-induced elevation of HGF/cMET signaling in normal mammary gland and cMET in tumors was significantly reversed with weight loss in C3(1)-Tag mice, with a concomitant and complete reversal of HFD-driven tumor progression (Sundaram et al. 2014b).

Given the precedent for the role of HGF signaling in invasive breast cancer (Gastaldi et al. 2010), a better understanding of HGF’s role in BBC tumorigenesis was necessary. We hypothesized that inhibition of cMET signaling through crizotinib therapy (PF-02341066) would reduce HFD-induced BBC. We first sought to inhibit tumor progression in existing tumors and began crizotinib treatment upon identification of the first palpable tumor. Crizotinib significantly reduced total tumor burden in both LFD- and HFD-fed C3(1)-Tag mice, with a corresponding reduction in microvascular density. We next investigated whether we could inhibit or delay tumorigenesis by treating C3(1)-Tag mice with crizotinib prior to tumor development. Crizotinib treatment paradoxically increased progression of the initially detected tumor in both diet groups. However, at sacrifice there were no differences between diet or treatment groups in total preneoplastic lesions, total tumor progression or tumor burden. In summary, cMET inhibition disrupted tumor vascularization and limited subsequent BBC tumor development in tumor-bearing mice. Our results suggest that reduction of microvascular density through cMET inhibition may be a viable therapeutic target in the treatment of BBC.

## Results

### Basal-like tumor latency was accelerated by HFD

**Fig. 1:**
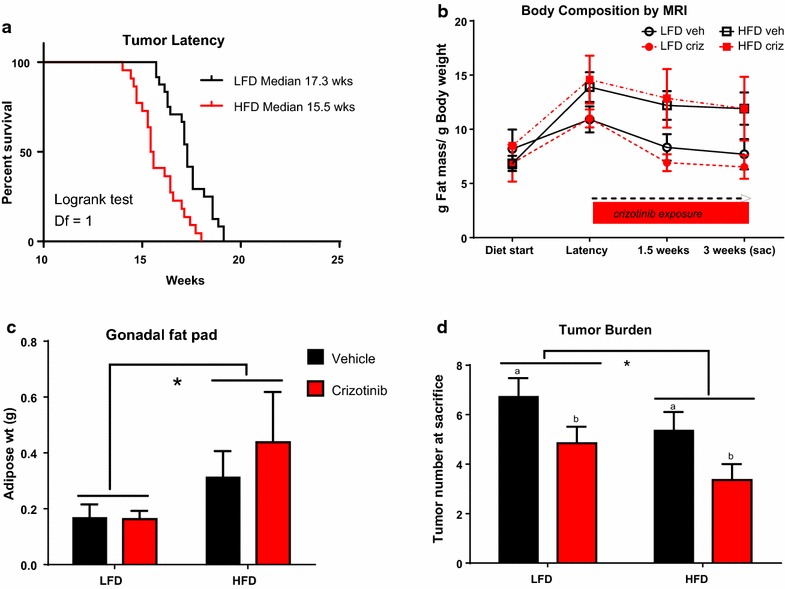
HFD accelerated tumor latency but reduced tumor burden, while crizotinib treatment blunted tumor burden regardless of diet. **a** Tumor latency was reported as age at detection of the first palpable tumor (LFD median 17.3 weeks, N = 24; HFD median 15.5 weeks, N = 22; P < 0.0001). **b** Body composition was measured at diet start, tumor latency (week 0 of crizotinib), 1.5 weeks on crizotinib treatment, and 3 weeks on crizotinib treatment (at sacrifice). **c** Gonadal fat pad mass was weighed at sacrifice (LFD vs. HFD N = 11–13 per group *P = 0.017). **d** Total number of visible tumors was assessed at sacrifice (LFD vs HFD *P = 0.0491; Vehicle (a) versus crizotinib (b) P = 0.0085)

Adult female C3(1)-Tag mice were randomly assigned to diet groups at 10 weeks of age (Additional file 1: Figure S1A, Model of Treatment study design) and body weight and body composition were monitored. Tumor latency and progression were tracked by palpation and calipers. Body weight was not significantly altered by diet or vehicle or drug treatment group for the study duration (Additional file 1: Figure S1B). Consistent with our previously reported results (Sundaram et al. 2013a), C3(1)-Tag mice fed HFD exhibited significantly decreased tumor latency compared with mice fed LFD (LFD median 17.3 weeks,; HFD median 15.5 weeks; P < 0.0001, Fig. 1a). Using a Chi square test with a degree of freedom of 1, LFD vs HFD equaled 15.72. Adiposity as measured by MRI was increased with HFD but did not reach significance (Fig. 1b). Oral gavage of vehicle or drug slightly reduced adiposity among all groups, irrespective of diet or treatment (Fig. 1b). Gonadal fat pad mass was significantly greater in mice fed HFD compared to mice fed LFD (*P* = 0.0173, Fig. 1c).

### Crizotinib treatment inhibited secondary tumor development

Crizotinib treatment significantly reduced total tumor burden, by 27.96 and 37.29 % in LFD- and HFD-fed C3(1)-Tag mice, respectively, compared to mice treated with vehicle (P = 0.0085, Fig. 1d). A minor diet effect on tumor burden was detected; HFD-fed animals in both vehicle- and crizotinib-treated groups showed reduced tumor burden at sacrifice compared to LFD-fed mice (P = 0.0491, Fig. 1d). Tumor progression of all palpable tumors was monitored using calipers throughout the 3-week measurement period between latency and sacrifice. No differences by diet or treatment group in primary tumor progression were detected (Additional file 1: Figure S1C).

### Crizotinib disrupted tumor vascularization

**Fig. 2:**
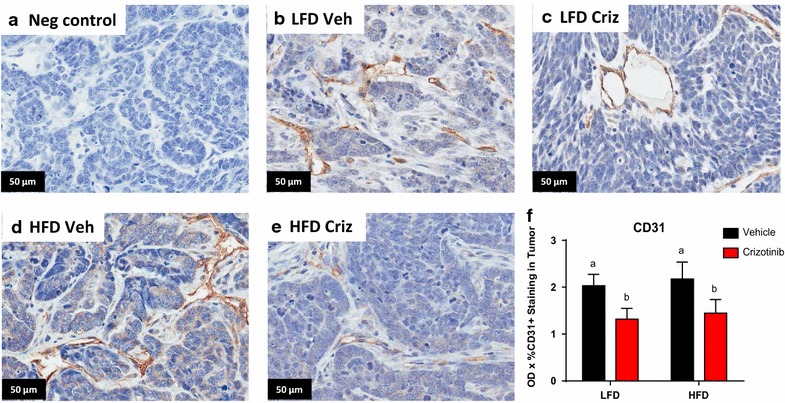
Crizotinib impaired tumor vascularization. **a**–**e** Representative photomicrographs (40×) of CD31 staining in negative control and indicated LFD, HFD, vehicle (veh) and crizotinib (criz) treated groups. **b** CD31 was quantified on 5–6 randomly selected regions of n = 2 sections each from each mouse. N = 9–10 mice (a vs b, Veh vs Criz, P = 0.0138)

CD31, a marker of angiogenic and lymphangiogenic microvessel density (MVD) (Choi et al. 2005), was measured by IHC (Fig. 2a–e). Crizotinib administration significantly reduced mean tumor MVD by 35.04 and 33.52 % in LFD and HFD groups, respectively (P = 0.014, Fig. 2f). There were no diet effects on CD31 positivity. Total cMET staining did not differ by diet or treatment group (Additional file 2: Figure S2A), while phosphorylated (active) cMET was significantly higher in mice fed HFD (P = 0.014, Additional file 2: Figure S2B).

### Crizotinib prophylactic treatment did not affect body weight or adiposity

**Fig. 3:**
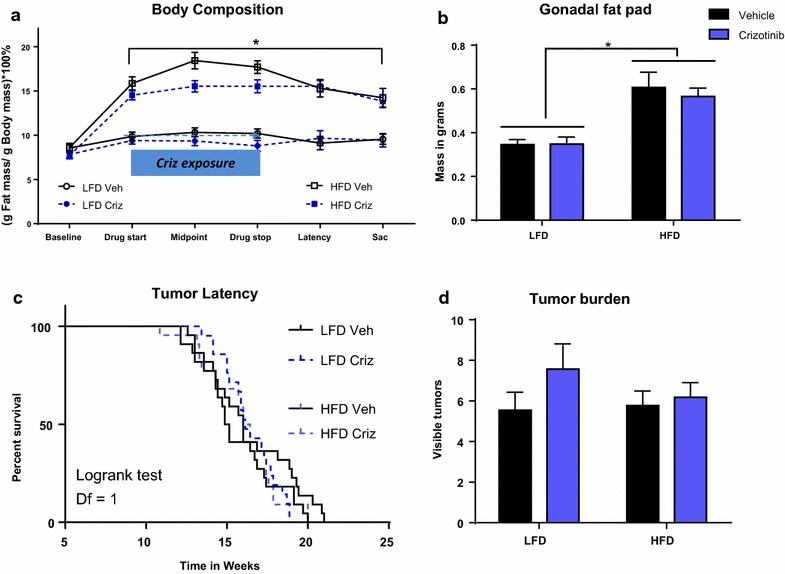
Preventive administration of crizotinib prior to tumor onset did not alter tumor parameters. Diet was started at 8 weeks of age (*baseline*). **a** Body composition was measured by MRI (LFD vs. HFD *P < 0.0001). **b** Gonadal fat pad mass was determined at sacrifice (LFD vs. HFD *P < 0.0001). **c** Upon initiation of crizotinib treatment (50 mg/kg by oral gavage) at 9 weeks of age, mice were palpated twice weekly for tumor onset. Median tumor latency did not differ with diet or treatment (LFD veh-treated median 15.0 weeks, N = 22; LFD criz-treated median 16.1 weeks, N = 21; HFD veh-treated median 16.0 weeks; HFD criz-treated median 16.2 weeks). **d** Total tumor burden was assessed at sacrifice

As crizotinib treatment reduced tumor burden in tumor-bearing mice, we next investigated whether we could inhibit or delay tumorigenesis by treating with crizotinib *prior to* tumor development. C3(1)-Tag tumors progress along the following timeline: atypical hyperplasia (AH) of the mammary ductal epithelium at 8 weeks of age, mammary intraepithelial neoplasia (resembling human carcinoma in situ [CIS]) at 12 weeks of age, and invasive carcinomas at 16 weeks of age with 100 % penetrance (Green et al. 2000). Thus, in the prevention arm of the study, mice were started on diet 2 weeks earlier than in the treatment study above to ensure crizotinib administration occurred within the primary window of AH/CIS precursor lesions. Starting at 8 weeks of age, mice were randomly assigned to LFD or HFD, with crizotinib treatment beginning at 9 weeks of age and continuing until 12 weeks of age (Additional file 3: Figure S3A). Mice fed HFD diet gained significantly more weight than the LFD mice, with greater body weights from 9 to 16 weeks of age (P  < 0.05, Additional file 3: Figure S3B). Body composition differed significantly between LFD- and HFD-fed mice beginning at 1 week on diet (9 weeks of age) and remained significant until sacrifice (P < 0.0001 for all data points, Fig. 3a). Crizotinib- and vehicle-treated mice fed HFD had significantly greater gonadal fat pad mass when compared to LFD-fed mice (P < 0.0001, Fig. 3b). No difference was detected in body weight or adiposity between the crizotinib and vehicle treated mice (Fig. 3a, b).

### Crizotinib prophylactic treatment increased tumor progression but did not significantly alter tumor burden or precursor lesions

Upon initiation of crizotinib or vehicle administration mice were palpated twice weekly for detection of tumor onset. Median tumor latency did not vary significantly between diet or treatment groups (LFD vehicle-treated median 15.0 weeks; LFD crizotinib-treated median 16.1 weeks; HFD vehicle median 16.0 weeks; HFD crizotinib median 16.2 weeks, Fig. 3c). Progression of the primary, initially detected tumor was significantly increased with crizotinib treatment in both diet groups (P = 0.04, Additional file 3: Figure S3C). However, when all tumors were considered there were no differences between diet or treatment groups in total tumor progression (Additional file 3: Figure S3D) or tumor burden (Fig. 3d) at sacrifice. Non-tumor mammary tissue was analyzed for AH and CIS premalignant lesions in of HFD-fed vehicle- or crizotinib-treated mice. There were no significant diet- or crizotinib-mediated effects on precursor lesion formation detected (Additional file 3: Figure S3E).

## Discussion

The HGF/cMET pathway is regulated by body weight and is relevant to BBC. HGF is the only known ligand for cMET, and the HGF/cMET signaling pathway has long been studied in normal development, ductal morphogenesis (Garner et al. 2011), invasive breast cancer (Tuck et al. 1996; Yamashita et al. 1994; Elliott et al. 2002; Wang et al. 1994; Jin et al. 1997; Beviglia et al. 1997), and invasive biology of several other cancers due to its angiogenic, mitogenic, and morphogenic effects (Stella et al. 2005; Sam et al. 2007). Notably, single nucleotide polymorphisms in the *MET* gene may be associated with metastatic breast cancer (Liu 2015). In patients, we demonstrated that 86 % of BBC expressed an HGF/cMET activation signature through gene expression (Casbas-Hernandez et al. 2013). Furthermore, a recent comprehensive meta-analysis including over 6000 cases showed that cMET over-expression is significantly associated with poor survival in breast cancer patients, particularly among patients with triple-negative breast cancer (Yan et al. 2015). Obesity mediates, and can exacerbate, both normal and tumor microenvironment dysfunction (Johnson et al. 2012; Sundaram et al. 2013b). HGF is an excellent candidate mediator of obesity-induced effects on cancer, as serum HGF is elevated in obese individuals and reduced with weight loss (Hiratsuka et al. 2005; Bell et al. 2006; Swierczynski et al. 2005). Moreover, adipose-derived HGF has been detected in normal and malignant breast tissue (Tuck et al. 1996).

We have previously demonstrated that HFD exposure during adulthood increased cMET expression and activation in normal mammary and tumors (Sundaram et al. 2013a). Diet-induced upregulation of cMET in tumors was also evident in mice fed HFD from weaning and could be reversed by weight loss (Sundaram et al. 2014b). Our previous work further revealed that HFD-induced weight gain resulted in increased mammary gland HGF that was reversed with weight loss (Sundaram et al. 2014b). Reduction in HGF due to weight loss correlated with diminished tumor progression or deceleration of tumor latency, depending on whether the diet was initiated at weaning or adult onset, respectively. Altogether, our previously published studies on HGF/cMET implicated HGF/cMET signaling as a mediator of obesity-driven BBC tumor aggression. Therefore, this study aimed to specifically inhibit cMET signaling as a potential BBC treatment or prevention strategy, hypothesizing that disruption of HGF/cMET signaling would mimic the effects of weight loss on BBC tumorigenesis.

C3(1)Tag mice are a GEMM that develop BBC in 100 % of female mice (Green et al. 2000). Initial characterization of the C3(1)-Tag model reported grossly palpable tumors at ~16 weeks of age; in our hands, median tumor latency occurs between 15 and 19 weeks, dependent on diet composition and age at diet start (Sundaram et al. 2013b, 2014a, b). Several groups have reported “windows of susceptibility” during which HFD and/or obesity may play a disproportionately greater role in promoting breast cancer onset (Biro and Deardorff 2013; Sundaram et al. 2013b). As shown here, in the crizotinib treatment arm of our study, initiating diets at 10 weeks of age resulted in detection of tumor latency two weeks earlier in mice that were fed HFD compared to mice fed LFD, consistent with our previous C(3)1-Tag studies in which identical diets were initiated at the same age (Sundaram et al. 2013a). However, in the crizotinib prevention arm of our study, in which HFD was initiated at 8 weeks of age, we did not see this diet effect of accelerated latency—these results parallel our second previously published study, in which female C3(1)-Tag mice were weaned onto LFD or HFD and no difference in latency was observed (Sundaram et al. 2014b). Interestingly, pubertal exposure to HFD has been linked to stunted mammary duct elongation and reduced mammary epithelial cell proliferation in murine models (Olson et al. 2010), a finding that was not seen in mice started on diet at 10 weeks of age or older. Collectively, our results here and in our previous studies support the concept that exposures extrinsic to the cancer cell (i.e., diet-induced alterations in the mammary microenvironment) can impact tumorigenesis, while age at diet start may be an important variable contributing to diet effects on tumor latency.

Herein, we showed that crizotinib treatment after BBC latency inhibited subsequent tumor formation such that total tumor burden was reduced at sacrifice, regardless of diet. The degree of tumor inhibition was paralleled by a similar degree of suppression of MVD, also irrespective of diet. Reduction in MVD in our crizotinib-treated tumor-bearing C3(1)-Tag mice could explain the significant reduction of tumor burden compared to vehicle-treated controls. In tumors, an “angiogenic switch” occurs, in which an increase in MVD in and near the tumor allows for tumor survival and metastasis (Lin and Pollard 2007). Increases in HGF in the tumor microenvironment contribute to this angiogenic switch (Wagatsuma et al. 1998; Abounader and Laterra 2005), while cMET signaling by the cancer cells facilitates invasion and migration away from the hypoxic interior of the tumor, entry into the new and leaky vessels, and metastasis to distant locations (Gastaldi et al. 2010). Indeed, obesity-promoted HGF production by fibroblasts, adipocytes, macrophages, and endothelial cells (Sundaram et al. 2013a, 2014b; Casbas-Hernandez et al. 2011, 2013; Mizuno and Nakamura 2013) may be a unique mechanism to increase blood vessel density and alleviate the hypoxia of obese adipose tissue. The fact that no changes were detected in latency in the prevention arm of our study suggests that early changes in the HGF/cMET axis (i.e., prior to invasive carcinoma) may not be as easily targeted, or are not causative in BBC tumorigenesis.

Initial mechanism-of-action studies for crizotinib showed that dose-dependent inhibition of cMET in gastric carcinoma, glioblastoma, and prostate carcinoma resulted in reduction of MVD as measured by CD31 (Zou et al. 2007). Inhibition of cMET as an effective anti-angiogenic agent has also been shown in xenograft models of aggressive cancers such as lung (Puri et al. 2007) and pancreatic cancers (You et al. 2011). Moreover, the use of other cMET inhibitors in triple-negative breast cancer models such as ours has yielded promising results in preclinical studies (Sameni et al. 2015). However, to date clinical trials using the cMET inhibitors tivantinib or onartuzamab in isolation or in combination with chemotherapy have demonstrated little therapeutic benefit in metastatic breast cancer (Tolaney et al. 2015; Dieras et al. 2015; Sharma and Adjei 2011). Clinical trials investigating crizotinib alone [ClinicalTrials.gov:NCT 02101385 (Schneider 2016)] or in combination with anti-VEGF therapy [ClinicalTrials.gov:NCT 02074878 (Baylor Breast Care Center 2015)] for the treatment of advanced triple negative breast cancer are currently underway. Breast cancer ranks as the fifth cause of death from cancer overall and is now the second cause of cancer death following lung cancer (Brewster et al. 2014; WHO 2014). With the growing global prevalence of obesity and the notable racial and ethnic disparities in BBC outcomes (Brewster et al. 2014), it is imperative that approaches are identified to effectively address the increased risk of breast cancer onset and progression to malignancy for an increasingly overweight and obese US population.

## Methods

### Antibodies and drugs

Crizotinib (PF-2341066 [(*R*)-3-[1-(2,6-dichloro-3-fluoro-phenyl)-ethoxy]-5-(1-piperidin-4-yl-1H-pyrazol-4-yl)-pyridin-2-ylamine) was purchased from Selleck Chemical (Catalog No. S1068). Primary antibodies include: Rabbit anti-mouse CD31 (Abcam #ab28364, Lot GR212364-5; 1:400); Rabbit anti-mouse phospho-cMET (Abcam #ab5662, Lot GR159296-1; 1:4000); Goat anti-mouse cMET (R&D Systems #AF527, Lot CTB0310091; 1:1000) diluted in Renoir Red Diluent (BM #PD904H). Additional reagents included biotin-conjugated Goat anti-rabbit IgG (Jackson #111-065-144, Lot 110335; 1:500); Donkey anti-goat IgG (Jackson #705-065-147, Lot 110544; 1:1000). ABC Elite (Vector #PK-6100, 1:50) and 3,3′ Diaminobenzidine (DAB) (Thermo Scientific #TA-125-QHDX).

### Animals and diet

Animal studies were performed with approval and in accordance with the guidelines of the Institutional Animal Care and Use Committee at the University of North Carolina at Chapel Hill. Animals were cared for according to the recommendations of the Panel on Euthanasia of the American Veterinary Medical Association. The veterinary care provided at UNC is in compliance with the Public Health Service Policy on Humane Care and Use of Laboratory Animals and meets the National Institutes of Health standards as set forth in the Guide for the Care and Use of Laboratory Animals (DHHS Publication No. (NIH) 85-23 Revised 1985). The animal facility is Association for Assessment and Accreditation of Laboratory Animal Care (AAALAC) approved and is responsible for the health and husbandry of animals. UNC also accepts as mandatory the PHS Policy on Humane Care and Use of Laboratory Animals be Awardee Institutions and NIH Principles for the Utilization and Care of Vertebrate Animals Used in Testing, Research, and Training. Animal studies comply with the ARRIVE guidelines. Mice were housed in a climate controlled Department of Laboratory Animal Medicine facility with a 12 h light:dark cycle and *ad libitium* access to food and water or special diets as defined below. Female C3(1)-Tag mice were obtained in collaboration with the UNC Lineberger Comprehensive Cancer Center (LCCC) Mouse Phase I Unit (MP1U). C3(1)-Tag mice (Green et al. 2000), a model of BBC, were generated by crossing heterozygous male mice with FVB/N non-transgenic female mice.

For the tumor treatment study, N = 46 female C3(1)-Tag mice were bred and maintained on chow diet (Harlan 2918) until nulliparous female were randomly assigned to LFD (N = 24) and HFD (N = 22) at 10 weeks of age. Diets obtained from Research Diets Inc. (New Brunswick, NJ, USA) were matched for protein, vitamins, and minerals, and provided 10 % kcal (“LFD”; # D11012202); and 60 % kcal (“HFD”; # D11012204) derived from fat. Diets were sucrose-free, and soy-free. Additional details of diet components are provided in Sundaram et al. (Sundaram et al. 2013a). Model of treatment study design is supplied in Additional file 1: Figure S1A). For the prevention study, during breeding and after weaning mice were put on Prolab Isopro RMH 3000 from LabDiet (St. Louis, MO, USA). At 8 weeks of age, nulliparous female C3(1)-Tag mice were randomly assigned to LFD (N = 43) and HFD (N = 45) diet groups (Model of treatment study design, Additional file 3: Figure S3A).

### Tumor latency, number, and progression

Mice were monitored for tumor development by palpating twice weekly. Tumor latency defined as age in weeks at detection of first tumor. Tumor volumes were measured twice weekly over 3 weeks using calipers to measure the width (short diameter) and length (long diameter) in millimeters for each tumor. Tumor volumes were calculated using the formula: length × width^2^ × 0.5. Tumor progression is reported as percent change in volume from latency to sacrifice 3 weeks later. Primary tumor progression refers to the first tumor identified; total tumor progression includes all tumors palpated. The total number of visible tumors per mouse was counted at sacrifice for total tumor burden.

### Crizotinib treatment

Crizotinib dosage was 50 mg crizotinib/kg of body weight (Zou et al. 2007). In the treatment study, crizotinib administration by oral gavage began at identification of the first palpable tumor and persisted for 3 weeks until sacrifice (5 days on drug, 2 days rest) (Additional file 1: Figure S1A). Briefly, drug was prepared by dissolving 20 mg of crizotinib powder in 200 µL 1 N hydrochloric acid (HCl), then brought to a total volume of 1 mL with vehicle (0.5 % glucose in phosphate-buffered saline) to yield a 2× crizotinib solution. Immediately prior to gavage administration, the 2x solution was diluted with an equal volume of vehicle to yield a 1x solution. In the prevention study, drug was prepared as a 1x solution by dissolving 10 mg of crizotinib powder in 200 µL 1 N hydrochloric acid (HCl), then brought to a total volume of 1 mL with vehicle; crizotinib treatment began for all mice at 9 weeks of age and continued for 3 weeks (5 days on drug, 2 days rest).

### Body weight and composition

Body weight was measured at start of diet and weekly until sacrifice. Body composition including lean mass, fat mass, free water content and total water content of non-anesthetized mice was measured using EchoMRI-100 quantitative magnetic resonance whole body composition analyzer (Echo Medical Systems, Houston, TX). Fat mass is presented as percent fat mass over total body weight (Sundaram et al. 2013a, 2014b). There were no significant changes in absolute lean mass in grams (data not shown).

### Tissue and blood collection

Three weeks after detection of the first palpable tumor, mice were fasted for 6 h and anesthetized by an intraperitoneal (i.p.) injection of avertin (tribromoethanol/amylene hydrate, 1.25 %) (Sigma Aldrich, St. Louis, MO). Blood was collected via cardiac puncture using an EDTA-coated syringe into 5 µL of 250 mM EDTA. Plasma was separated from other blood components by centrifugation at 10,000×*g* for 2 min at 4 °C. Mammary tumors, unaffected inguinal mammary gland, liver, spleen, and lungs were flash frozen in liquid nitrogen or were placed into a cassette and formalin-fixed for immunohistochemistry (IHC) and H&E analysis. All frozen samples were stored at −80 °C until analyzed.

### Immunohistochemistry

Briefly, formalin-fixed and paraffin-embedded tissues were sectioned at 5 microns and mounted for histological staining (Sundaram et al. 2013a). Tissues were baked, deparaffinized, and hydrated. Following heat-induced epitope retrieval (Rodent Decloaker BM#RD913L), slides were treated with 3 % hydrogen peroxide in de-ionized water. Tissues were treated with Avidin/Biotin Block (Vector #SP-2001) and exposed to primary antibodies (anti-CC3, anti-Ki67 anti-CD31; anti-phospho-cMET; anti-cMET) diluted in Renoir Red Diluent at 4 °C overnight. Following incubation with biotin-conjugated secondary antibodies [Goat anti-rabbit IgG; Donkey anti-goat IgG] tissue sections were treated with ABC Elite and DAB. Digital immunohistochemistry quantification was performed following the protocol previously described in Sundaram et al. (2013a). Stained slides were scanned into the Aperio Scanscope CS system (Aperio Technologies, Vista, CA, USA) at a magnification of 40× and were quantified using the Aperio Imagescope software. Scanned slides were analyzed using algorithms as described previously (Sundaram et al. 2014a). N  =  5–6 random areas from sections (n  =  2 sections per mouse) were quantified and averaged per tumor per animal (n  =  9–10 mice per diet or exposure group). Images shown are representative.

### Statistical analysis

Data are expressed as mean ± standard error of the mean (SEM). All means were compared by 2 way analysis of variance (ANOVA) with Tukey’s post hoc test for statistical differences using GraphPad Prism 5 software (GraphPad Software, Inc. La Jolla, CA). Kaplan–Meier analyses were conducted using GraphPad Prism 5 software to estimate tumor latency. Log rank and Chi square tests were used to investigate differences among groups. P values <0.05 were considered statistically significant.

## Additional files

10.1186/s40064-016-1920-3 A) Model of Treatment Study design. At 10 weeks of age female mice were randomized to LFD or HFD and palpation began for identification of tumor onset. Vehicle or crizotinib treatment by oral gavage began at detection of first palpable tumor and lasted for 3 weeks, 5 days on and 2 days off. Mice were sacrificed at 3 weeks past tumor onset. Metabolic and other parameters were measured as indicated. B) Body weights did not differ by diet or treatment group. No significant differences were observed in C) primary or D) total tumor progression.
10.1186/s40064-016-1920-3 A) Total MET staining did not differ by diet or treatment group. B) Phosphorylated (active) MET was significantly higher in mice fed HFD (2-way ANOVA *P = 0.0141).
10.1186/s40064-016-1920-3 A) Model of Prevention Study design. At 8 weeks of age female mice were randomized to LFD or HFD. At 9 weeks of age vehicle or crizotinib treatment by oral gavage began and lasted for 3 weeks, 5 days on and 2 days off. Palpation began for identification of tumor onset also began at 9 weeks of age. Mice were sacrificed at 3 weeks past tumor onset. Metabolic and other parameters were measured as indicated. B) Mice fed HFD diet gained significantly more weight than the LFD mice, with greater body weights from 9 to 16 weeks of age (LFD vs HFD *P  < 0.05). Progression of the primary tumor was significantly increased with crizotinib treatment in both diet groups (a vs b, P = 0.036), but had no effect on total tumor progression or total tumor burden.
