# Supplementary figures and images for: cMET inhibitor crizotinib impairs angiogenesis and reduces tumor burden in the C3(1)-Tag model of basal-like breast cancer
Source: Springerplus. 2016 Mar 19;5:348. doi: 10.1186/s40064-016-1920-3 (PMC4799044; doi:10.1186/s40064-016-1920-3)

# Additional File 1: Figure Supplement 1

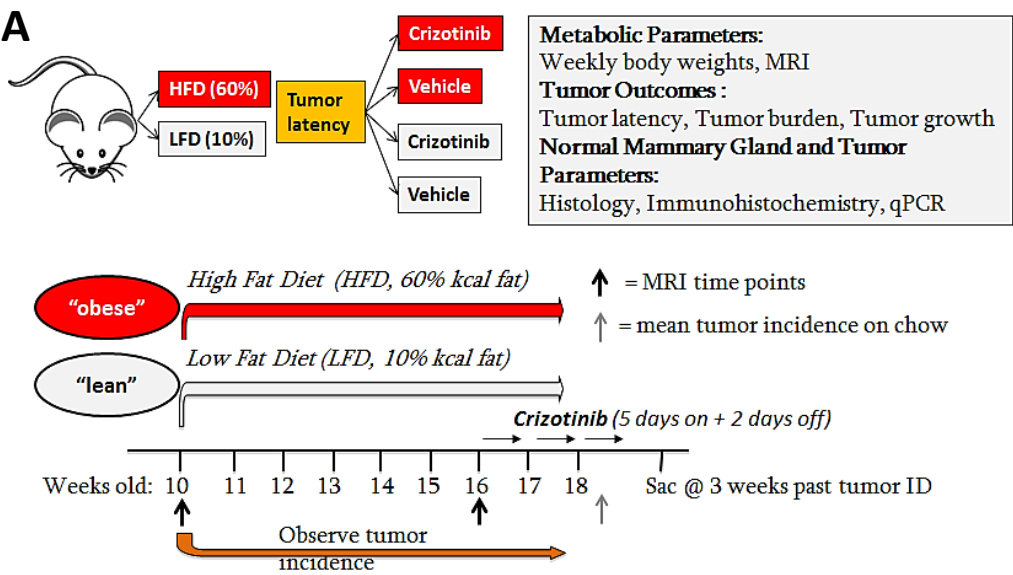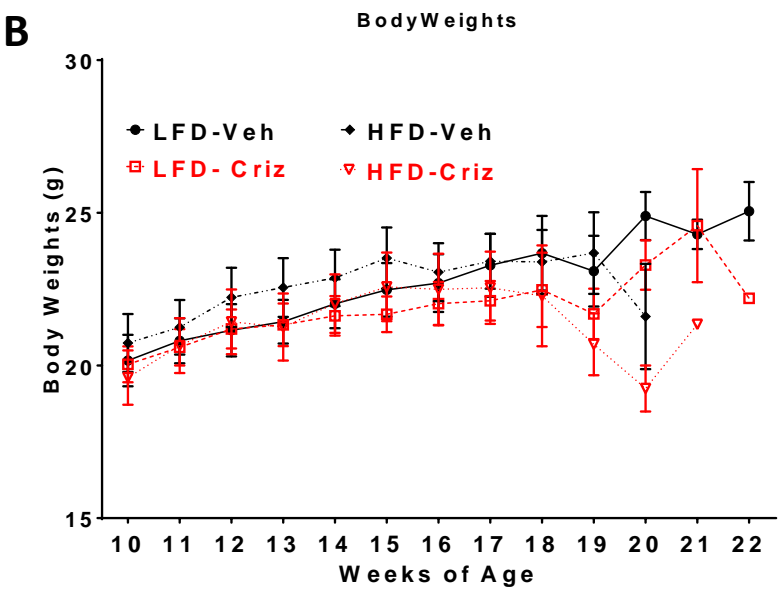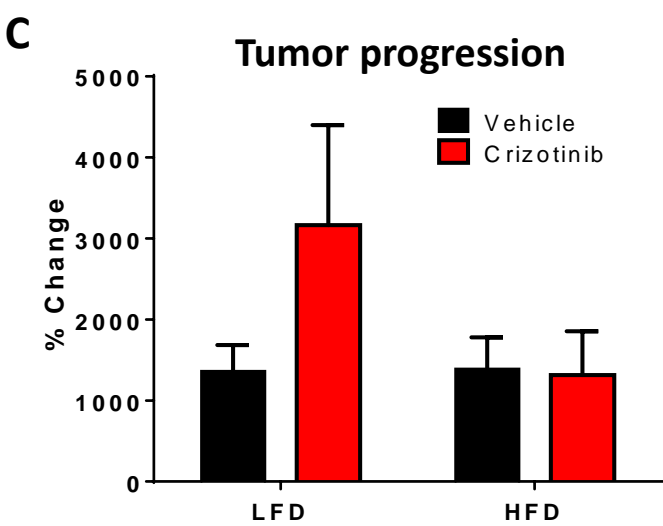

Supplement: Supplementary file 1 — 10.1186/s40064-016-1920-3 A) Model of Treatment Study design. At 10 weeks of age female mice were randomized to LFD or HFD and palpation began for identification of tumor onset. Vehicle or crizotinib treatment by oral gavage began at detection of first palpable tumor and lasted for 3 weeks, 5 days on and 2 days off. Mice were sacrificed at 3 weeks past tumor onset. Metabolic and other parameters were measured as indicated. B) Body weights did not differ by diet or treatment group. No significant differences were observed in C) primary or D) total tumor progression. [file 40064_2016_1920_MOESM1_ESM.pdf]

Additional File 2: Figure Supplement 2

A

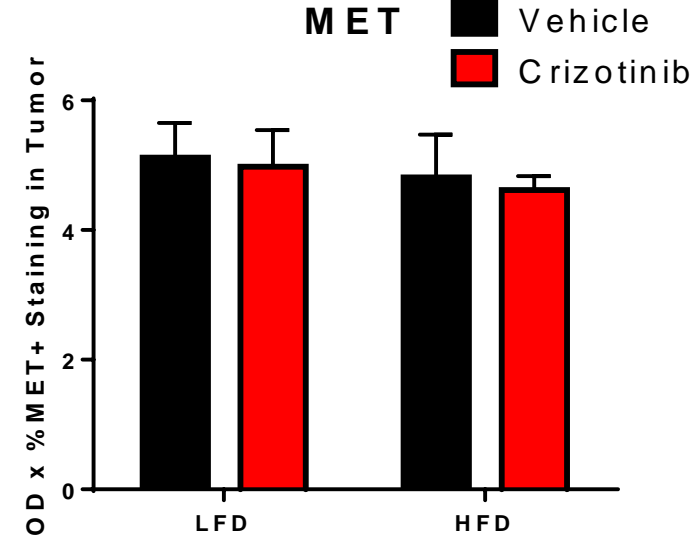

B

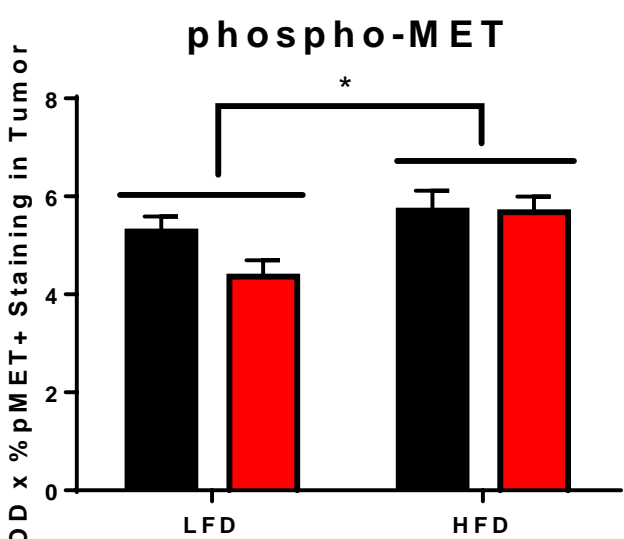

Supplement: Supplementary file 2 — 10.1186/s40064-016-1920-3 A) Total MET staining did not differ by diet or treatment group. B) Phosphorylated (active) MET was significantly higher in mice fed HFD (2-way ANOVA *P = 0.0141). [file 40064_2016_1920_MOESM2_ESM.pdf]

Additional File 3: : Figure Supplement 3

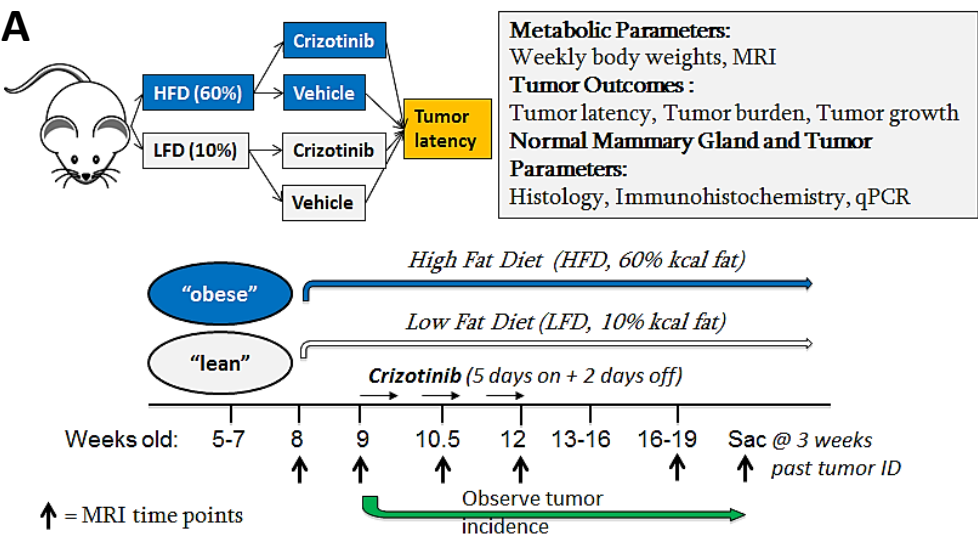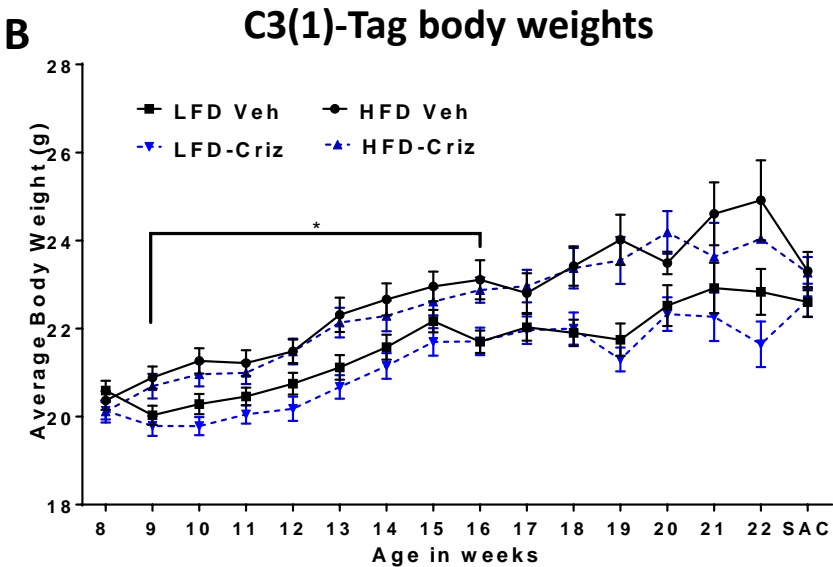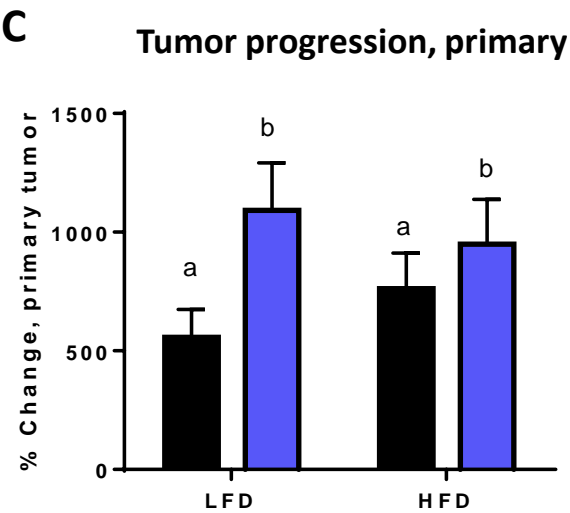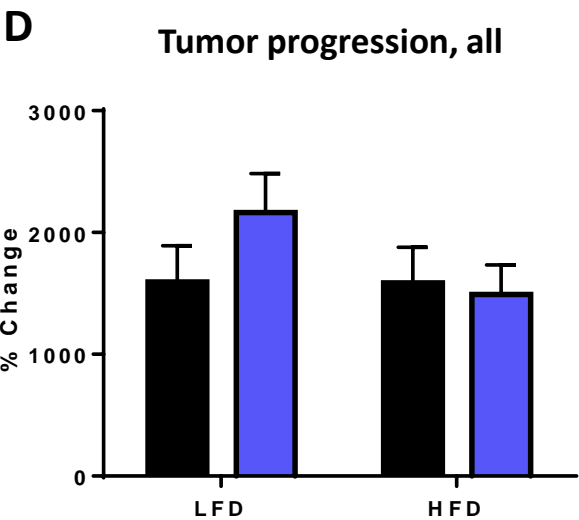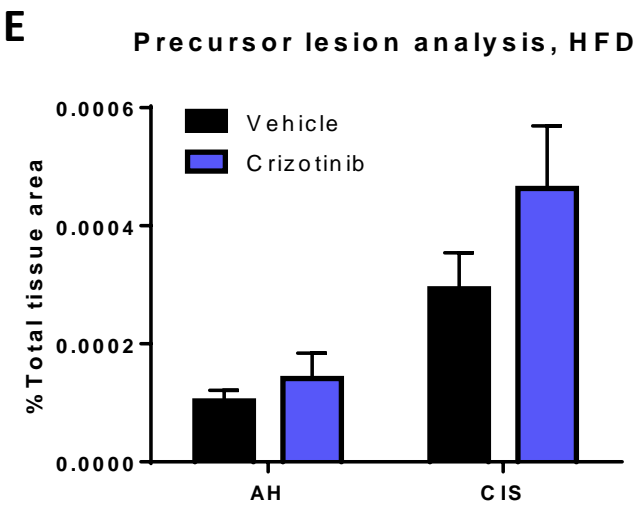

Supplement: Supplementary file 3 — 10.1186/s40064-016-1920-3 A) Model of Prevention Study design. At 8 weeks of age female mice were randomized to LFD or HFD. At 9 weeks of age vehicle or crizotinib treatment by oral gavage began and lasted for 3 weeks, 5 days on and 2 days off. Palpation began for identification of tumor onset also began at 9 weeks of age. Mice were sacrificed at 3 weeks past tumor onset. Metabolic and other parameters were measured as indicated. B) Mice fed HFD diet gained significantly more weight than the LFD mice, with greater body weights from 9 to 16 weeks of age (LFD vs HFD *P < 0.05). Progression of the primary tumor was significantly increased with crizotinib treatment in both diet groups (a vs b, P = 0.036), but had no effect on total tumor progression or total tumor burden. [file 40064_2016_1920_MOESM3_ESM.pdf]
